# Microarray profiling of long noncoding RNAs associated with idiopathic pulmonary arterial hypertension

**DOI:** 10.3892/etm.2019.7783

**Published:** 2019-07-17

**Authors:** Bing Han, Peili Bu, Xiao Meng, Xiaoyang Hou

Exp Ther Med 13: 2657-2666, 2017; DOI: 10.3892/etm.2017.4355

Subsequently to the publication of the above article, the authors discovered numerous mistakes in the manuscript that had arisen as a consequence of rearrangement of the data. These problems with the data led to a dispute among the authors concerning the copyright of the article, leading later to the authors’ unanimous decision to retract this article from the Journal. The authors apologize to the readership of the Journal for any inconvenience caused.

